# A meta-analysis of the vascular endothelial growth factor polymorphisms associated with the risk of pre-eclampsia

**DOI:** 10.1042/BSR20190209

**Published:** 2020-05-20

**Authors:** Weicheng Duan, Chenlu Xia, Kang Wang, Yijie Duan, Ping Cheng, Bo Xiong

**Affiliations:** 1 Department of Forensic Medicine, Tongji Medical College, Huazhong University of Science and Technology, Wuhan 430030, P.R. China; 2 Department of Gynecology, The Central Hospital of Wuhan, Tongji Medical College, Huazhong University of Science and Technology, Wuhan 430014, P.R. China

**Keywords:** meta-analysis, polymorphisms, pre-eclampsia, risk, VEGF

## Abstract

**Backgroud:** Pre-eclampsia (PE) is a common pregnancy-induced hypertension disease. Some case–control studies reported the association between vascular endothelial growth factor (VEGF) gene polymorphisms (rs3025039, rs2010963) and PE risk. However, these associations were inconsistent in several studies. Therefore, we conducted this meta-analysis to assess the role of VEGF gene polymorphisms in PE more precisely.

**Methods:** Eligible studies were searched in PubMed, Embase, Web of Science and Chinese (Chinese National Knowledge Infrastructure (CNKI) and WanFang) databases. Statistical analyses were performed by Stata 12.0 software. Odds ratio (OR) and 95% confidence interval (CI) were used to assess the strength of the association. In addition, subgroup analyses, sensitive analyses and publication bias analyses were performed to further assess this meta-analysis.

**Results:** In total, 21 studies were included in the meta-analysis covering 2018 cases and 2632 controls. There were significant associations between VEGF polymorphisms (rs3025039, rs2010963) and PE risk in the overall populations. In the subgroup analyses, we found that rs3025039 polymorphism was associated with the increased risk of PE among Chinese. As for rs2010963 polymorphism, a significant association was observed in subgroup of Caucasian.

**Conclusion:** The present study suggested that the two VEGF gene polymorphisms (rs3025039, rs2010963) are associated with increased risk of PE in different ethnic groups, which means that the targets may be useful genetic markers for early prediction of PE.

## Introduction

Pre-eclampsia (PE), a common pregnancy disease diagnosed by hypertension and proteinuria, occurs in approximately 2–8% of pregnancies [1,2]. PE is an important reason for the maternal and fetal morbidity and mortality due to dysfunction of multiple systems and organs, such as liver, kidney and brain [3]. Although its etiology has not been well recognized, PE is now regarded as the result of the combined effect of multiple factors [4,5]. According to the results of the several epidemiological studies, PE has a substantial heritable component, which is estimated to be a major effect [4,6].

The vascular endothelial growth factor (VEGF) gene, located on chromosome 6p21.3, is a key regulator of angiogenesis and vascular function. Therefore, VEGF is vital for the formation of trophoblasts, embryonic vasculature and maternal and foetal blood cells *in utero* [7]. Abnormal vascular growth and endothelial dysfunction have been proposed to be the part of pathogenesis. Hence, VEGF has drawn the attention of many researchers [8,9].

The associations between polymorphisms of the VEGF gene and PE have been extensively studied [10–30]. However, the results were somewhat controversial. In 2013, two meta-analyses assessed the associations among four polymorphisms of the VEGF gene and the risk of PE [31,32]. But the retrieved datasets of these two meta-analyses were not sufficient, and several new studies have been published regarding this relationship between VEGF gene polymorphisms (rs3025039, rs2010963) and PE [10–15]. In addition, the results published recently remained inconsistent and conflicting, likely owing to heterogeneity of different researches or inadequate sample size. A comprehensive retrieval of the pertinent literature in multiple databases is likely to help assess disease risks more precisely. In view of the mortality of PE, more efficient biomarkers are required for early discovery and prevention in the clinical practice. Therefore, we performed an updated meta-analysis of all eligible studies including English and non-English journals to investigate the association between VEGF gene polymorphisms and the risk of PE. Moreover, we further divided the cases by ethnic groups, countries as well as genotyping methods and analyzed subgroup specific associations.

## Methods

### Identification of literature

This meta‐analysis was conducted in accordance with the guidance of the Preferred Reporting Items for Systematic Reviews and Meta‐Analyses (PRISMA) statement [33]. The PRISMA Checklist was presented in Supplementary Table S1. The literature search using the electronic databases PubMed, EMBASE, Web of Science, Chinese National Knowledge Infrastructure (CNKI) and WanFang was conducted by two study investigators. The comprehensive search strategies included the Mesh term and Keywords: (‘vascular endothelial growth factor’ or ‘VEGF’), (‘polymorphism’, ‘SNP’ ‘variant’, ‘genotype’ or ‘mutations’), (‘Pre-eclampsia’, ‘Preeclampsia’, ‘Pregnancy Toxemias’, ‘Pregnancy Toxemia’, ‘Edema-Proteinuria-Hypertension Gestosis’, ‘Edema Proteinuria Hypertension Gestosis’, ‘EPH Complex’, ‘EPH Toxemias’, ‘EPH Toxemia’, ‘Proteinuria-Edema-Hypertension Gestosis’ or ‘Proteinuria Edema Hypertension Gestosis’) through 3 January 2019. All eligible studies were retrieved and examined carefully. Review articles and references of other relevant researches were further searched to find additional eligible studies.

### Inclusion and exclusion criteria

The inclusion criteria were as follows: (i) studies which estimated the associations between VEGF rs2010963 or rs3025039 and the susceptibility to PE; (ii) case–control studies or cohort studies of PE; (iii) patients must be clinically diagnosed for PE (blood pressure ≥ 140/90 mmHg on two measurements with ≥1+ proteinuria or 300 mg/24 h after the 20th week of pregnancy); (iv) reported the allele frequencies of both cases and controls for different genotypes; (v) genotype distribution in the control group confirmed by Hardy–Weinberg equilibrium (HWE). The exclusion criteria of the meta-analysis were: (i) non-human studies, meta-analysis, comments, letters, reviews, mechanism studies or studies without controls, (ii) studies with overlapping or incomplete data. When overlapped population between studies was identified, only the newest or most complete article was included in the analysis. According to the corresponding criteria, two independent authors screened the articles.

### Data extraction and assessment of methodological quality

Data were extracted by two authors independently from each study. The following information was collected: first author, publication year, participants’ country, ethnicity (categorized as Caucasian, Xanthoderm, Indo-European hybrid), sample size, study design (case–control or cohort), genotyping method, alleles and genotype frequency distribution in cases and controls, and the major conclusion of the study. When incomplete or apparent conflicting data were found in the article, we made an attempt to contact authors. Inconsistencies in data interpretation were resolved with discussion. The Newcastle–Ottawa Quality Assessment Scale (NOS) was employed to evaluate the methodological quality of the identified articles, and scores ranging from 0 (the worst) to 9 (the best) were assigned based on the quality of the studies. The studies with no less than 5 stars were considered to be of high quality.

### Statistical analysis

First, deviation from HWE in the distribution of allele frequencies was estimated again by the chi-square test (determined by *P*<0.05). Stata 12.0 was used to perform quantitative meta-analysis. The association was estimated with four models: Allele comparison model, Dominant model, Recessive model and Homozygote model. The four models of the data analysis were conducted by the random-effects model to prevent exaggerated results. The association between the VEGF rs3025039 or rs2010963 and PE risk was assessed by the raw odds ratios (ORs) with 95% confidence intervals (CIs). The Student’s *t* test was used to determine the significance of the crude OR, and *P*<0.05 was considered statistically significant. In addition, heterogeneity assumption among the included researches was evaluated by the Chi-square and *I^2^*, which was regarded to be statistically significant if *P*<0.10. And *I^2^* values of 25, 50 and 75% were nominally assigned as low, moderate and high estimates. To insure that any single study did not cause an obvious influence to the whole effects, sensitivity analysis was performed to estimate the validity and stability of the study. In addition, to further analyze the source of the heterogeneity and the specific association between the VEGF polymorphism and PE, studies were also divided into several subgroups on the basis of the country, the ethnicity of the related population and the genotyping method. Egger’s test was performed to estimate the potential publication bias.

## Results

### Study characteristics

**Figure 1:**
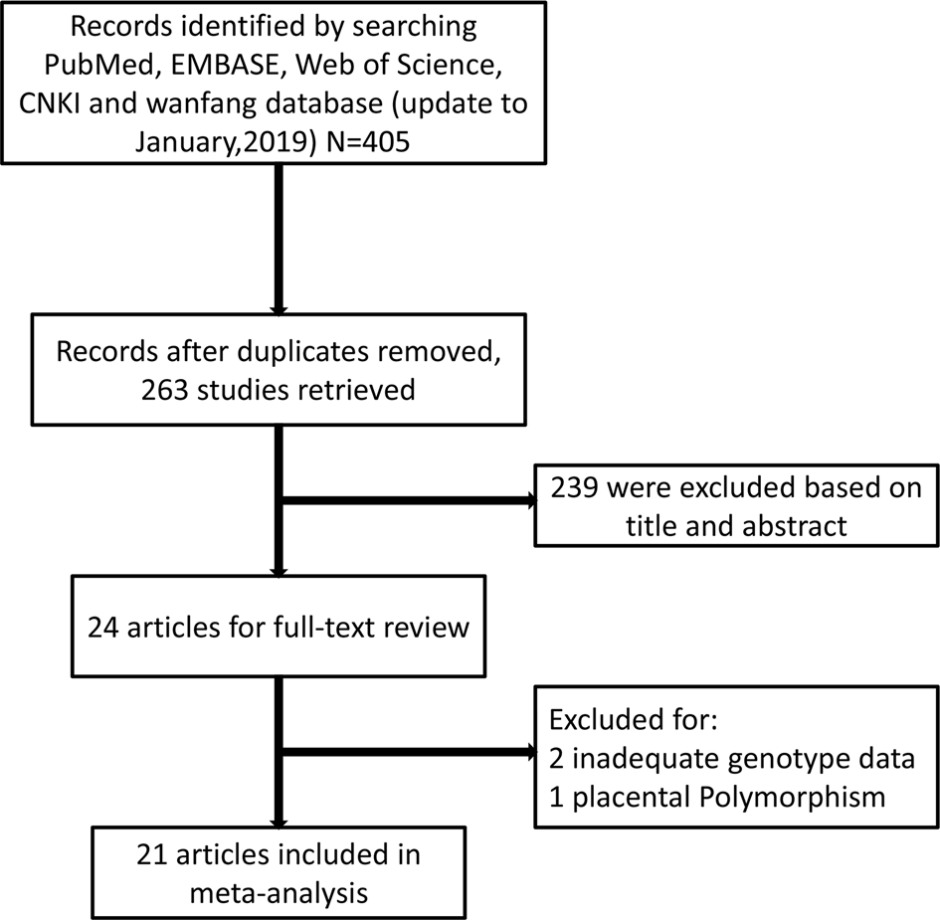
PRISMA flow chart of selection procedure

As shown in Figure 1, the PRISMA flowchart demonstrated process of the literature retrieval. Four hundred and five studies were identified according to the result of the retrieval strategy and manual searches from PubMed, EMBASE, Web of Science, CNKI and WanFang database. On the basis of our inclusion/exclusion criteria, 142 studies were excluded for duplication and 239 studies were excluded as meta-analysis, reviews, mechanism studies or non-relevant research. Then, 24 studied were selected for full-text review. However, three studies were excluded, because two studies lacked genotype data and one study focused on placental polymorphism. Because most of the studies did not use the rs number to name the SNP, every SNP was manually confirmed by searching in the NCBI according to the sequence in the literature. Finally, 21 studies were included in the meta-analysis. Thereinto, 15 studies assessed the association between VEGF rs3025039 T/C polymorphism and the risk of PE, and 12 studies examined the association between VEGF rs2010963 C/G polymorphism and the risk of PE. The specific information about the included studies was exhibited in Table 1. The quality evaluation of each study following the NOS is presented in Table 2, which showed all of these studies can be regarded as high-quality studies.

**Table 1:** Study characteristics of PE cases and controls in the analysis of VEGF polymorphisms

Author [Ref.]	Year	Country	Ethnicity	Sources of controls	Number		Polymorphism(s)	Genotyping method	Association findings
					Case	CTR			
Lu [10]	2017	China	Xanthoderm	HB	156	286	rs3025039	Snapshot	NS
Amosco [11]	2016	Philippines	Xanthoderm	HB	165	191	rs3025039 rs2010963	MassARRAY system	Supportive NS
Salimi [12]	2015	Iran	Caucasian	HB	192	186	rs2010963	PCR-RFLP	Supportive
Silva [13]	2015	Brazil	Caucasian	HB	79	210	rs2010963	PCR-RFLP	NS
Zhang Honghui [14]	2014	China	Xanthoderm	HB	58	70	rs3025039 rs2010963	Sequencing	Supportive NS
Procopciuc [15]	2014	Romania	Caucasian	HB	70	94	rs3025039	PCR-RFLP	Supportive
Chedraui [16]	2013	Ecuador	Indo-European hybrid	HB	31	31	rs3025039 rs2010963	Sequencing	NS
Andraweere [17]	2013	Australia	Caucasian	HB	174	168	rs3025039	MassARRAY system	NS
Atis [18]	2012	Turkey	Caucasian	HB	34	58	rs3025039	MassARRAY system	NS
Chen Baoli [19]	2011	China	Xanthoderm	HB	84	71	rs3025039	PCR-RFLP	Supportive
He Yun [20]	2011	China	Xanthoderm	HB	61	43	rs3025039 rs2010963	Sequencing	Supportive
Garza-Veloz [21]	2011	Mexico	Indo-European hybrid	HB	86	78	rs2010963	PCR-RFLP	NS
Cunha [22]	2010	Brazil	Caucasian	HB	52	28	rs3025039	PCR-RFLP	NS
Liu Shifang [23]	2010	China	Xanthoderm	HB	84	71	rs3025039	PCR-RFLP	Supportive
Huang Yuliang [24]	2009	China	Xanthoderm	HB	128	231	rs3025039	PCR-RFLP	Supportive
Sandrim [25]	2008	Brazil	Caucasian	HB	94	108	rs2010963	TaqMan-assays	NS
Nagy [26]	2008	Hungary	Caucasian	HB	71	93	rs2010963	real-time PCR	NS
Shim [27]	2007	Korea	Xanthoderm	HB	110	209	rs3025039	PCR-RFLP	Supportive
Kim [28]	2007	Korea	Xanthoderm	HB	223	237	rs3025039 rs2010963	Snapshot	NS
Banyasz [29]	2006	Hungary	Caucasian	HB	84	96	rs2010963	PCR-RFLP	Supportive
Papazoglou [30]	2004	Sweden	Caucasian	HB	42	73	rs3025039 rs2010963	PCR-RFLP	Supportive NS

Abbreviations: CTR, control; HB: hospital-based study; NS: non-significant.

**Table 2:** Quality assessment conducted according to the Newcastle–Ottawa Scale for all the included studies

	Selection				Comparability	Exposure			
Author	Adequate definition of case	Representativeness of the cases	Selection of controls	Definition of controls	Comparability of cases and controls	Exposure assessment	Same method of ascertainment for cases and controls	Non-response rate	Total score
Lu [10]	*	*		*		*	*	*	6
Amosco [11]	*	*		*	*	*	*	*	7
Salimi [12]	*	*		*	*	*	*	*	7
Silva [13]	*	*		*	*	*	*	*	7
Zhang Honghui	*	*		*		*	*		5
Procopciuc	*	*		*	*	*	*	*	7
Chedraui	*	*		*	*	*	*	*	7
Andraweere	*	*		*	*	*	*	*	7
Atis	*	*		*	*	*	*	*	7
Chen Baoli	*	*		*		*	*		5
He Yun	*	*		*		*	*	*	6
Garza-Veloz	*	*		*	**	*	*	*	8
Cunha	*	*		*	*	*	*	*	7
Liu Shifang	*	*		*	*	*	*		6
Huang Yuliang	*	*		*		*	*		5
Sandrim	*	*		*	**	*	*	*	8
Nagy	*	*		*	*	*	*	*	7
Shim	*	*		*	**	*	*	*	8
Kim	*	*		*	*	*	*	*	7
Banyasz	*	*		*	**	*	*	*	8
Papazoglou	*	*		*	**	*	*	*	8

### Overall analysis

**Figure 2:**
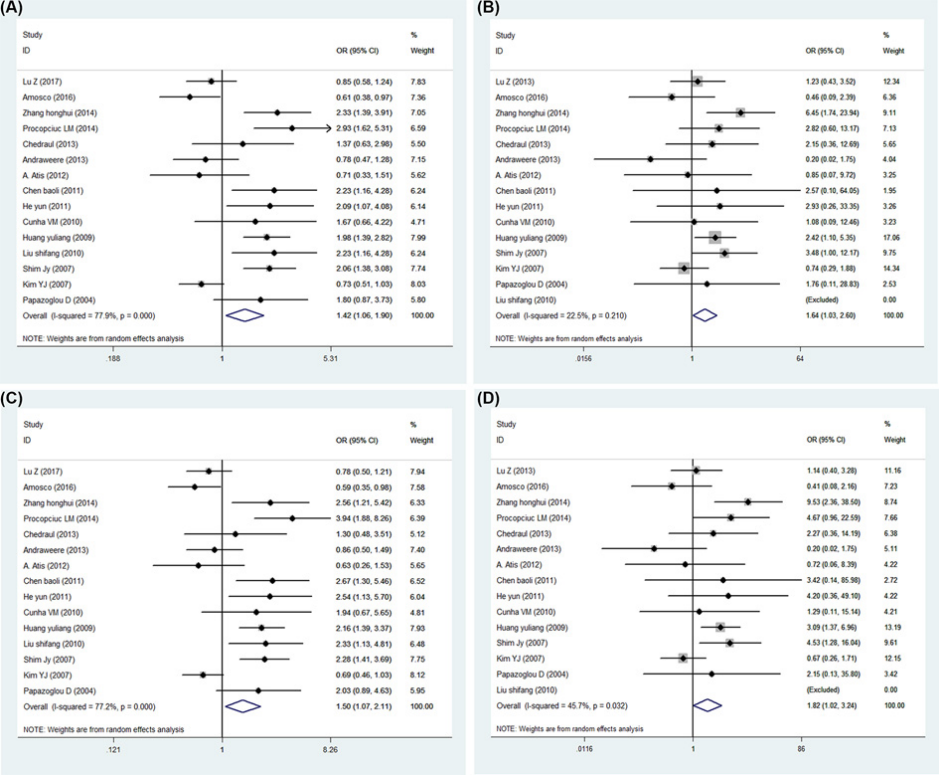
Forest plot of PE risk associated with VEGF gene rs3025039 polymorphism (**A**) Allele comparison model. (**B**) Dominant model. (**C**) Recessive model. (**D**) Homozygote model.

**Figure 3:**
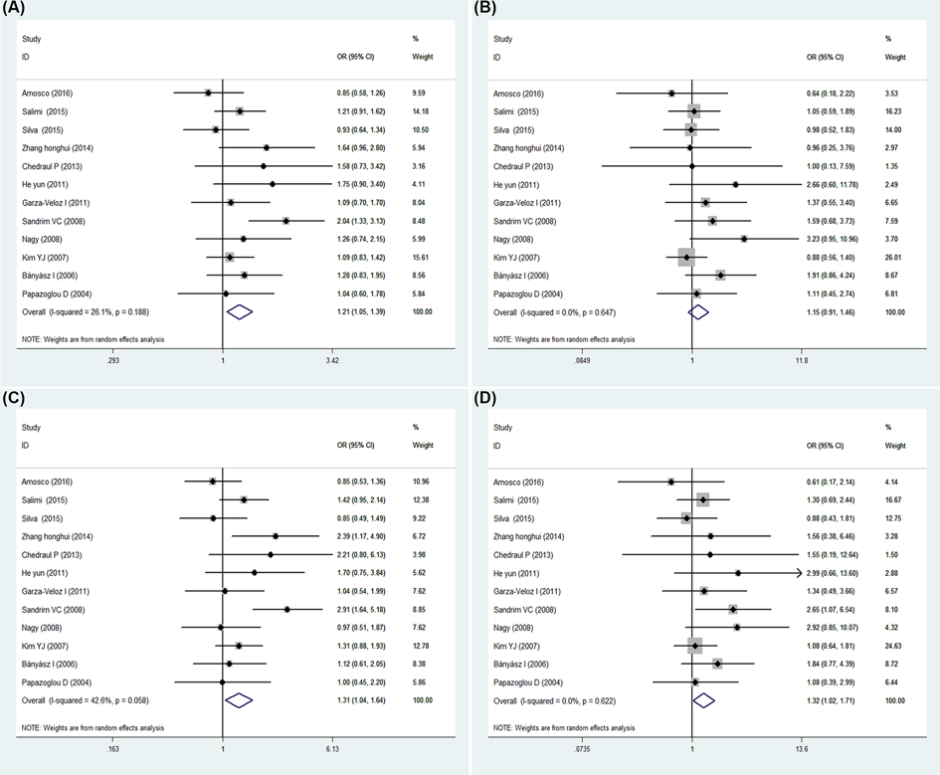
Forest plot of PE risk associated with VEGF gene rs2010963 polymorphism (**A**) Allele comparison model. (**B**) Dominant model. (**C**) Recessive model. (**D**) Homozygote model.

Overall results of this meta-analysis between the two SNP and PE are displayed in Tables 3 and 4. In total, we analyzed 1426 cases and 1872 controls for rs3025039 with the random-effect model, showing a significantly increased risk for the comparison of the T allele to the C allele (OR = 1.418, 95% CI = 1.060–1.898, *P*=0.019, Figure 2A). Also, the results of the three genotype models analysis all revealed a significant association between PE and the VEGF rs3025039 (Dominant model: OR = 1.637, 95% CI = 1.031–2.598, *P*=0.037, Figure 2B; Recessive model: OR = 1.501, 95% CI = 1.068–2.109, *P*=0.019, Figure 2C; Homozygote model: OR = 1.819, 95% CI = 1.021–3.240, *P*=0.042, Figure 2D), in which the result of the Recessive model exhibited high heterogeneity (*I^2^* = 77.2%) and others were acceptable. An analysis of 1148 cases and 1388 controls for rs2010963 showed the C allele in allele comparison model, CC and CG genotype in the recessive model and CC genotype in the homozygous model increased the risk of PE significantly (Allele comparison model: OR = 1.207, 95% CI = 1.046–1.394, *P*=0.010, Figure 3A; Recessive model: OR = 1.310, 95% CI = 1.044–1.643, *P*=0.020 Figure 3C; Homozygote model: OR = 1.324, 95% CI = 1.024–1.713, *P*=0.032, Figure 3D), however the result of the dominant model did not indicate statistical significance (OR = 1.154, 95% CI = 0.912–1.460, *P*=0.232, Figure 3B).

**Table 3:** Main results for the rs3025039 polymorphism with the risk of PE

Comparison	Subgroup	Number	Test of association			Test of heterogeneity	
			OR	95% CI	*P*-value	*I^2^*	*P*-value
T vs C	Overall	15	1.418	1.060–1.898	**0.019**	76.6%	<0.001
	China	6	1.793	1.229–2.617	**<0.001**	69.5%	0.006
	Korea	2	1.219	0.438–3.392	0.704	93.3%	<0.001
	Other Countries	7	1.189	0.738–1.917	0.476	73.6%	<0.001
	Xanthoderm	9	1.454	0.995–2.125	**0.053**	83.5%	<0.001
	Caucasian	5	1.361	0.758–2.444	0.301	73.1%	0.005
	Indo-European hybrid	1	1.369	0.628–2.984	0.429	/	/
	MassARRAY system	2	0.681	0.484–0.958	**0.028**	<0.1%	0.483
	Sequencing	2	1.942	1.182–3.191	**0.009**	20.0%	0.264
	PCR-RFLP	9	1.961	1.594–2.413	**<0.001**	14.1%	0.316
	Snapshot	2	0.778	0.601–1.007	0.056	<0.1%	0.566
TT vs CC+CT	Overall	14	1.637	1.031–2.598	**0.037**	22.5%	0.210
	China	5	2.420	1.400–4.180	**0.002**	<0.1%	0.436
	Korea	2	1.514	0.334–6.873	0.591	73.6%	0.052
	Other countries	7	1.062	0.502–2.246	0.911	<0.1%	0.465
	Xanthoderm	9	1.795	0.979–3.291	0.059	42.1%	0.098
	Caucasian	5	1.144	0.438–2.987	0.784	1.0%	0.400
	Indo-European hybrid	1	2.148	0.364–12.693	0.399	/	/
	MassARRAY system	2	0.338	0.091–1.256	0.105	<0.1%	0.555
	Sequencing	2	4.378	1.525–12.572	**0.006**	<0.1%	0.328
	PCR-RFLP	8	2.409	1.399–4.150	**0.002**	<0.1%	0.980
	Snapshot	2	0.926	0.461–1.858	0.828	<0.1%	0.478
TT +CT vs CC	Overall	15	1.501	1.068–2.109	**0.019**	75.1%	<0.001
	China	6	1.933	1.205–3.102	**0.006**	71.6%	<0.001
	Korea	2	1.243	0.385–4.019	0.716	92.9%	<0.001
	Other countries	7	1.260	0.724–2.193	0.414	73.6%	<0.001
	Xanthoderm	9	1.520	0.977–2.365	0.063	82.8%	<0.001
	Caucasian	5	1.513	0.769–2.977	0.230	72.9%	0.005
	Indo-European hybrid	1	1.295	0.477–3.515	0.612	/	/
	MassARRAY system	2	0.700	0.481–1.019	0.063	<0.1%	0.317
	Sequencing	2	1.976	1.035–3.772	**0.039**	12.1%	0.286
	PCR-RFLP	9	2.186	1.678–2.848	**<0.001**	24.0%	0.230
	Snapshot	2	0.726	0.540–0.978	**0.035**	<0.1%	0.697
TT vs CC	Overall	14	1.819	1.021–3.240	**0.042**	45.7%	0.032
	China	5	3.009	1.403–6.453	**0.005**	32.3%	0.206
	Korea	2	1.658	0.256–10.750	0.596	82.3%	0.017
	Other countries	7	1.139	0.467–2.781	0.775	24.9%	0.239
	Xanthoderm	9	2.041	0.958–4.346	0.064	60.6%	0.013
	Caucasian	5	1.256	0.376–4.198	0.711	32.2%	0.207
	Indo-European hybrid	1	2.267	0.362–14.185	0.382	/	/
	MassARRAY system	2	0.316	0.085–1.177	0.086	<0.1%	0.605
	Sequencing	2	5.284	1.322–21.116	**0.019**	33.0%	0.222
	PCR-RFLP	8	3.120	1.793–5.429	**<0.001**	<0.1%	0.921
	Snapshot	2	0.846	0.420–1.707	0.641	<0.1%	0.462

Presentation with bold indicated a statistical significance.

**Table 4:** Main results for the rs2010963 polymorphism with the risk of PE

Comparison	Subgroup	Number	Test of association			Test of heterogeneity	
			OR	95% CI	*P*-value	*I^2^*	*P*-value
C vs G	Overall	12	1.207	1.046–1.394	**0.010**	26.1%	0.188
	Xanthoderm	4	1.178	0.879–1.581	0.273	46.1%	0.135
	Caucasian	6	1.246	1.004–1.546	**0.046**	38.4%	0.150
	Indo-European hybrid	2	1.199	0.817–1.760	0.353	<0.1%	0.417
	Sequencing	2	1.620	1.044–2.516	**0.032**	<0.1%	0.940
	PCR-RFLP	6	1.149	0.971–1.358	0.105	<0.1%	0.640
	Other methods	4	1.224	0.866–1.731	0.253	68.1%	0.024
CC vs GG+GC	Overall	12	1.154	0.912–1.460	0.232	<0.1%	0.647
	Xanthoderm	4	0.932	0.626–1.387	0.729	<0.1%	0.510
	Caucasian	6	1.295	0.948–1.768	0.104	<0.1%	0.462
	Indo-European hybrid	2	1.296	0.564–2.975	0.541	<0.1%	0.784
	Sequencing	2	0.974	0.314–3.020	0.964	<0.1%	0.976
	PCR-RFLP	6	1.231	0.896–1.690	0.200	<0.1%	0.689
	Other methods	4	1.203	0.676–2.142	0.529	43.2%	0.152
CC +GC vs GG	Overall	12	1.310	1.044–1.643	**0.020**	42.6%	0.058
	Xanthoderm	4	1.350	0.898–2.031	0.149	52.3%	0.098
	Caucasian	6	1.278	0.894–1.826	0.178	56.0%	0.045
	Indo-European hybrid	2	1.360	0.667–2.772	0.397	33.4%	0.221
	Sequencing	2	2.328	1.294–4.189	**0.005**	<0.1%	0.901
	PCR-RFLP	6	1.174	0.924–1.491	0.189	<0.1%	0.663
	Other methods	4	1.322	0.804–2.175	0.272	73.8%	0.010
CC vs GG	Overall	12	1.324	1.024–1.713	**0.032**	<0.1%	0.622
	Xanthoderm	4	1.134	0.734–1.753	0.570	<0.1%	0.433
	Caucasian	6	1.461	1.019–2.094	**0.039**	8.9%	0.359
	Indo-European hybrid	2	1.377	0.557–3.406	0.489	<0.1%	0.905
	Sequencing	2	1.555	0.479–5.048	0.462	<0.1%	0.994
	PCR-RFLP	6	1.287	0.907–1.827	0.157	<0.1%	0.699
	Other methods	4	1.469	0.771–2.799	0.242	49.0%	0.117

Presentation with bold indicated a statistical significance.

### Subgroup analyses

The subgroup analyses were carried out due to the heterogeneity of result and biases of the different subgroups. The results of subgroup analyses were shown in Tables 3 and 4. First of all, the different countries of population were divided into three parts including China, Korea and other countries in rs3025039 according to the source of the population in studies. For countries subgroup analyses in rs3025039 polymorphism, a significant correlation was found in the allele model and the three genotype models of the Chinese subgroup, in which the Allele comparison model showed moderate heterogeneity (*I^2^* = 69.5%), the Dominant model showed low heterogeneity (*I^2^* < 0.001%), the Recessive model showed high heterogeneity (*I^2^* = 71.6%) and the Homozygote model showed moderate heterogeneity (*I^2^* = 32.3%). However, no significant association was found in the Korea’s subgroup and other countries’ subgroup, and relatively high heterogeneity was observed in the almost all models of the two subgroups except in Dominant and Homozygote model of other countries’ subgroup, which indicated the differences of population countries were not the major cause of the heterogeneity for rs3025039 in this meta-analysis. Then, we performed an ethnic restriction including Xanthoderm, Caucasian and Indo-European hybrid for further subgroup analysis in rs3025039. No significant association was observed in the any model, and the heterogeneity of various models in different subgroups showed no significant reduction. Next, subgroup analysis was performed in rs3025039 according to the genotyping methods including MassARRAY system, Sequencing, PCR-RFLP and Snapshot. A significantly increased risk was found in the Sequencing and PCR-RFLP subgroup, but a protective effect was found in the MassARRAY system subgroup with Allele comparison model and Snapshot subgroup with Recessive model. The heterogeneity of all the subgroup in the four models were relatively low, which indicated that the genotyping methods might be the major source of the heterogeneity.

For rs2010963, we performed the subgroup analysis based on ethnicity and genotyping methods, because the overlapping countries were limited. The subgroups of ethnicity were divided as the same as the subgroups of rs3025039. A significant association was observed in the Caucasian subgroup with both Allele comparison model and Homozygote model (Allele comparison model: OR = 1.246, *P*=0.046; Homozygote model: OR = 1.461, *P*=0.039), and other subgroups showed no obvious difference. Moreover, the heterogeneity of allele comparison model and homozygote model in Caucasian subgroup was low (Allele comparison model: *I^2^* = 38.4%, Homozygote model: *I^2^* = 8.9%). However, the difference between the overall heterogeneity and subgroup heterogeneity was not apparent, illustrating that the ethnicity was not the important cause of heterogeneity in the meta-analysis. Regarding the genotyping methods subgroups, we divided into three groups: Sequencing, PCR-RFLP and other methods, owing to the duplicating number of the genotyping methods. The result of the Sequencing subgroup showed a statistically significant in Allele comparison model and Homozygote model with indistinctive heterogeneity (Allele comparison model: OR = 1.620, *P*=0.032, *I^2^* < 0.1%; Recessive model: OR = 2.328, *P*=0.005, *I^2^* < 0.1%). Besides, heterogeneity of the Sequencing and PCR-RFLP subgroups in all models was not significant (*I^2^* < 0.1%), while heterogeneity of other methods subgroups was higher. The results suggested that the source of the heterogeneity might be the genotyping methods, consistent with the conclusion above.

### Sensitivity analysis and publication bias

**Figure 4:**
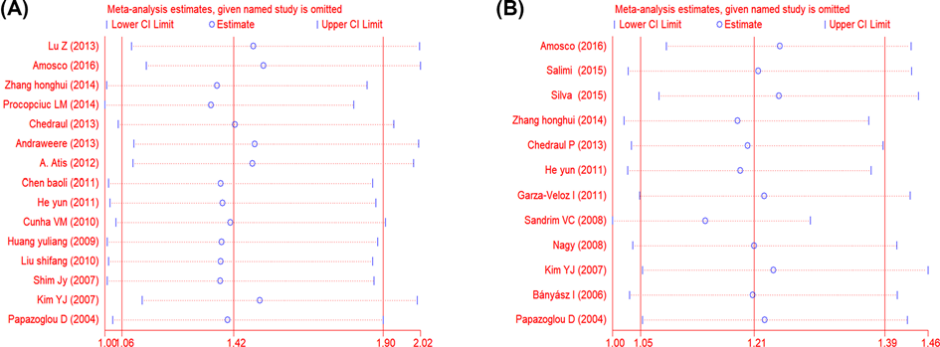
Sensitive analyses of individual study for VEGF gene polymorphisms (**A**) rs3025039 polymorphism. (**B**) rs2010963 polymorphism.

To confirm the reliability of our results, a sensitivity analysis was performed for the allele model, showing no apparent difference before and after the removal of each study shown in Figure 4. In addition, publication bias assessed by Egger’s regression test present no obvious evidence in statistics, which was displayed in Table 5.

**Table 5:** Egger’s regression test of the two VEGF polymorphisms

		rs3025039				rs2010963			
		T vs C	TT vs CC+CT	TT+CT vs CC	TT vs CC	C vs G	CC vs GG+GC	CC+GC vs GG	CC vs GG
Egger’s test	*P-*value	0.319	0.678	0.126	0.847	0.182	0.153	0.518	0.227
	95% CI	[−2.12, 6.02]	[−2.05, 1.38]	[−0.91, 6.60]	[−2.26, 1.88]	[−0.88, 4.05]	[−0.43, 2.40]	[−2.30 4.27]	[−0.67 2.50]

## Discussion

Although the etiology of PE is considered to be multifactorial, genetic factors are thought to be strong determinants of this disease [4,6]. Early studies reported that VEGF genes were associated with vascular growth and endothelial dysfunction, which may somewhat interpret the development of PE. In recent decades, many researchers have been focusing on the role that VEGF gene may play in the cause of PE [34]. However, case–control studies have shown contradictory associations between VEGF gene polymorphisms and PE. The aim of this meta-analysis was to evaluate the association between VEGF rs3025039 and rs2010963 polymorphisms and PE for the use of the biomarkers in the clinical practice and the investigation of the concrete pathomachanism.

We conducted a thorough literature retrieve and review to identify as many relevant studies as possible in our meta-analysis. Compared with previous meta-analyses, we made an effort to gain some improvements in our analysis: first, several studies were not included in previous meta-analyses (Lum (2017), Amosco et al. (2016), Salimi et al. (2015), Silva et al. (2014), Zhang Honghui et al. (2014), Procopciuc et al. (2014), Atis et al. (2012), Chen Baoli et al. (2011), Liu Shifang (2010)); second, multiple subgroups were divided to be analyzed; thereby a more adequate statistical power was gained in our study. Similar to the published researches, we found significant associations between the two VEGF gene polymorphisms (rs3025039, rs2010963) and PE, suggesting VEGF gene variants in rs3025039 and rs2010963 loci might be involved in the development of PE. Our results provide evidence of a significantly increased risk about rs3025039 polymorphisms for PE with the four models. Compared with the previous meta-analyses, a significantly increased risk for PE was observed in rs2010963 polymorphisms with less heterogeneity except for the Dominant model. In the stratified analysis by ethnicity and countries for rs3025039, a significantly increased risk of pre-eclampsia was observed in studies conducted among Chinese population. As for subgroup analyses of ethnicity in rs2010963, a statistically association was found in the Allele comparison and the Homozygote models of Caucasian.

In addition, the heterogeneity could be accounted for by the subgroup analysis of genotyping methods. For rs3025039 polymorphism, the subgroup analyses of four genotyping methods including MassARRAY system, Sequencing, PCR-RFLP and Snapshot all showed low levels of heterogeneity (*I^2^* < 40%, *P*>0.10), where the results of the PCR-RFLP and Sequencing were consistent with the total result (OR > 1, *P*<0.05), but different from the result of the other genotyping methods. Similarly, the heterogeneity of the three subgroups covering PCR-RFLP and Sequencing and other methods was different for rs2010963 polymorphism. The heterogeneity of the PCR-RFLP and Sequencing subgroup was quite low (*I^2^* < 10%, *P*>0.1), whereas the heterogeneity of the other methods subgroup was extensive (*I^2^* > 40%). The reason could be that studies in each subgroup are relatively few or different genotyping methods may influence the genotyping result. This observation is similar to previous studies, in which differences in genotyping methods might contribute to heterogeneity [35,36]. The results would be more reliable and accurate if the same appropriate genotyping method was applied in different studies, because different genotyping methods have specialty in different aspects. Genotyping results with new genotyping technologies need to be confirmed using direct sequencing. Furthermore, we have made efforts to seek out the potential sources of heterogeneity via sensitivity analysis assess and publication biases assessment through Egger’s test, demonstrating impact of the individual literature and the publication biases were not obvious. Although the exact pathogenesis of how the SNPs change VEGF and PE susceptibility are not fully understood, a significant correlation between VEGF SNPs (rs3025039 and rs2010963) and PE have been confirmed by our present meta-analysis. At present, several biomarkers have been associated with PE, including soluble endoglin, Flt-1, MAP, PlGF and so on [37–39]. Integration of more reliable biomarkers and figuring out the feasibility in different ethnical groups will increase the accuracy the prediction of the PE, which is quite important for the early prevention of PE. In the present study, our results provide the evidence that the status of the VEGF is close to occurrence of PE and the two SNPs of the VEGF could be applied in prediction of PE, particularly different ethnical groups.

Several limitations of our meta-analysis should be acknowledged. First, unpublished reports or studies published in other non-international journals could not be included in the analysis. These problems may have affected the stability of the meta-analysis data. Secondthe pooled sample sizes for the subgroup analyses among Xanthoderm and Caucasian for both rs2010963, rs3025039 were relatively small (<2000 for cases), which may limit the statistical power. Third, the recruitment criteria of patients and controls varied in different studies. Finally, gene–gene or gene–environment interactions were not considered in the present study—such as age, smoking, alcohol status and mental state—which may have influenced the associations between VEGF gene polymorphisms and PE risk. Nevertheless, this meta-analysis improves our understanding of the associations between two polymorphisms of VEGF gene and the risk of PE.

In conclusion, the two VEGF gene polymorphisms are associated with an increased risk of pre-eclampsia in different ethnic groups, respectively. A large number of and high-quality studies are required to establish more precise evidence and minimize the bias in meta-analysis.

## Supplementary Material

Supplementary Table S1

## Abbreviations

CI: confidence interval
CNKI: Chinese National Knowledge Infrastructure
Flt-1: fms-related tyrosine kinase 1
HWE: Hardy–Weinberg equilibrium
MAP: mitogen-activated protein
NOS: Newcastle–Ottawa Quality Assessment Scale
OR: odds ratio
PE: pre-eclampsia
PIGF: placental growth factor
PRISMA: Preferred Reporting Items for Systematic Reviews and Meta‐Analysis
SNP: single nucleotide polymorphism
VEGF: vascular endothelial growth factor
